# The Usefulness of Adaptative Radiotherapy in Prostate Cancer: How, When, and Who?

**DOI:** 10.3390/biomedicines10061401

**Published:** 2022-06-13

**Authors:** Rodrigo Muelas-Soria, Rafael García-Mollá, Virginia Morillo-Macías, Jorge Bonaque-Alandí, Patricia Sorribes-Carreras, Francisco García-Piñón, Carlos Ferrer-Albiach

**Affiliations:** 1 Department of Radiation Oncology, Hospital Provincial de Castellón, Avda. Dr Clará 19, 12002 Castellón de la Plana, Spain; vmorill@gmail.com (V.M.-M.); ferreralbiach@gmail.com (C.F.-A.); 2 Department of Medical Physics and Radioprotection, Hospital General de Valencia, Av. de las Tres Cruces, 2, 46014 Valencia, Spain; rafagarciamo@gmail.com; 3 Department of Medical Physics and Radioprotection, Hospital Provincial de Castellón, Avda. Dr Clará 19, 12002 Castellón de la Plana, Spain; jorgebonaque@gmail.com; 4 Department of Dietetics and Nutrition, Hospital Provincial de Castellón, Avda. Dr Clará 19, 12002 Castellón de la Plana, Spain; patsorribes@yahoo.es; 5 Department of Biostatistics, Fundación Hospital Provincial de Castellón, Avda. Dr Clará 19, 12002 Castellón de la Plana, Spain; fgp.ctm@gmail.com

**Keywords:** prostate cancer, adaptive radiotherapy, deformable image registration

## Abstract

The aim of this study was to develop a deformable image registration (DIR)-based offline ART protocol capable of identifying significant dosimetric changes in the first treatment fractions to determine when adaptive replanning is needed. A total of 240 images (24 planning CT (pCT) and 216 kilovoltage cone-beam CT (CBCT)) were prospectively acquired from 24 patients with prostate adenocarcinoma during the first three weeks of their treatment (76 Gy in 38 fractions). This set of images was used to plan a hypofractionated virtual treatment (57.3 Gy in 15 fractions); correlation with the DIR of pCT and each CBCT allowed to translate planned doses to each CBCT, and finally mapped back to the pCT to compare with those actually administered. In 37.5% of patients, doses administered in 50% of the rectum (D50) would have exceeded the dose limitation to 50% of the rectum (R50). We first observed a significant variation of the planned rectal volume in the CBCTs of fractions 1, 3, and 5. Then, we found a significant relationship between the D50 accumulated in fractions 1, 3, and 5 and the lack of compliance with the R50. Finally, we found that a D50 variation rate [100 × (administered D50 − planned D50/planned D50)] > 1% in fraction three can reliably identify variations in administered doses that will lead to exceeding rectal dose constraint.

## 1. Introduction

The acquisition of kilovoltage cone-beam CT (CBCT) is currently considered the standard of care in prostate image-guided radiotherapy (IGRT) [1]. It allows the visualization of the prostate and nearby organs at risk (OARs) down to a geometric accuracy level of approximately 1 mm and enables the 3D registration of soft tissues by planning CT (pCT) [2]. Some anatomical variations after pCT cannot be corrected by IGRT alone and result in clinically significant changes in the administered doses [3], and, if hypofractionated scheme treatments are used, become amplified, because each fraction represents a higher proportion of the overall dose [4,5].

Adaptive radiotherapy (ART) techniques have been developed to ensure the correctness of treatment delivery to the prostate and OARs [6]. Offline ART strategies are the most commonly applied [7], which use CBCT images to determine potential anatomical variations with respect to the pCT and study how they may influence the planned treatment [8]. Thus, physicians can decide whether treatment plans should be adapted to the real conditions.

Offline procedures use information gathered during the first few fractions to make some assumptions about forthcoming behavior (statistical prediction) for individual patients [6]. Some of them are limited because they are based on the comparison of planned and administered doses of single fractions [9,10]. Deformable image registration (DIR) algorithms have provided a technical solution to this limitation, as they take into account anatomical variations and allow the calculation of accumulated doses administered to the tumor and the OARs for any fraction of the treatment [11]. Indeed, its usefulness has already been demonstrated for treating prostate tumors [12].

The biggest challenge we may find when using offline ART techniques is not to re-plan treatment, but to identify as early as possible when the treatment being administered differs significantly from the planned treatment. For this reason, the purpose of this study was to develop a DIR-based offline ART protocol capable of identifying significant dosimetric changes in the first treatment fractions so to determine when adaptive replanning is necessary. 

## 2. Materials and Methods

### 2.1. Study Subjects

Twenty-four patients with intermediate or high-risk prostate adenocarcinoma submitted for treatment with our 3D-IGRT protocol [13] (76 Gy in 38 fractions) and androgen deprivation therapy, between February 2014 and February 2016, were prospectively enrolled. All the patients underwent a physical examination, prostate biopsy, pretreatment prostate-specific antigen analysis, bone scan, and multiparametric magnetic resonance imaging. This study was approved by the institutional ethics committee, and all patients signed informed consent.

### 2.2. Simulation and Contouring 

One week before the pCT, the patients started a mildly laxative diet combined with a laxative drug (macrogol) and an antiflatulent agent (simethicone). A 150 mL enema was administered on both the night and morning before the test. Patients were asked to completely void their bladder and drink 600 cc of plain water 30 min before the scan. The pCT was performed in the supine position with knee and foot support. Images were acquired with a Siemens SOMATOM Sensation 16 CT scanner (Siemens AG, Erlangen, Germany), with intravenous contrast, and using a 3 mm slice thickness and 1 mm pixel size. When a rectal diameter exceeding 3 cm was observed, the patients were rescanned after the implementation of rectal emptying strategies [14].

Two CTVs were used: the CTV57 (the prostate gland including any extracapsular spread, and the entirety of the involved seminal vesicles [SVs]), and the CTV45 (the complete SVs, even when these were not involved). The planning target volume (PTV) margins for both these CTVs was 1 cm superior, 0.5 cm inferior and posterior, and 0.8 cm anterior and right–left. The rectal circumference was contoured as an OAR from the rectosigmoid flexure to the beginning of the anal canal. The entire bladder and the femoral heads were also contoured.

### 2.3. Virtual Planning

Patients were treated with standard fractionation (76 Gy in 38 fractions), but a virtual VMAT plan was created to assess the need to adapt the treatment plan with a hypofractionated schedule. It is in these treatments where the evaluation and possible adaptation of the original treatment plan is critical. We generated a virtual hypofractionated VMAT (volumetric-modulated arc therapy) schedule to deliver 57.3 Gy in 15 fractions with a PTV57 (57 Gy, 3.82 Gy/fraction; 2 Gy equivalent dose with α/β = 1.5 Gy [EQD2_1,5_] = 87.1 Gy) and a PTV45 (45 Gy, 3 Gy/fraction; EQD2_1,5_ = 57.86 Gy). The planning objectives were that at least 98% of PTV57 would receive 98% of the prescribed dose (D98 ≥ 98%) and that the 2% of PTV57 would not exceed 107% of the prescribed dose (D2 ≤ 107%). The OARs dose constraints were calculated to be equivalent to the QUANTEC recommendations [15]: 20% and 50% of the rectum volume, respectively, could receive 51 Gy and 36.5 Gy (V51 < 20% and V36.5 < 50%), while the D2 would receive less than 99% of the prescribed dose. Moreover, we allowed 35% of the bladder volume to receive up to 57 Gy (V57 < 35%).

### 2.4. Image-Guided Treatment Control 

Seven days before starting the treatment, and until its end, the patients resumed the intestinal preparation protocol. During the treatment they also followed the bladder-filling protocol. The patients were positioned pretreatment using an Elekta Synergy™ XVI image-guided radiotherapy-capable CBCT linear accelerator imaging system (Crawley, UK; release 4.2.1). A positioning CBCT scan was performed daily for automatic fusion with the pCT, with a pelvic collimator to completely visualize the pelvic structures. Rectal diameters exceeding 3 cm were not tolerated.

### 2.5. Adaptive Radiotherapy Process 

In this study we used nine nonconsecutive CBCT images (Monday, Wednesday, and Friday) recorded during the first three weeks of each patient’s treatment (a total of 216 CBCTs). We assumed that the doses delivered on Tuesday and Thursday were identical to those given the following day.

We used the RayStation treatment planning station (TPS; v.4.5, RaySearch Laboratories AB, Stockholm, Sweden) hybrid DIR in our workflow to correlate the pCT and all the CBCT images. The reliability of this algorithm has already been validated for prostate cancer [12]. This DIR allow mapping pCT structures to each CBCT and dose in the opposite direction, from each CBCT to pCT. This allowed us to calculate the accumulated dose in the pCT at any time during the treatment: an essential factor in offline ART [16].

**Figure 1:**
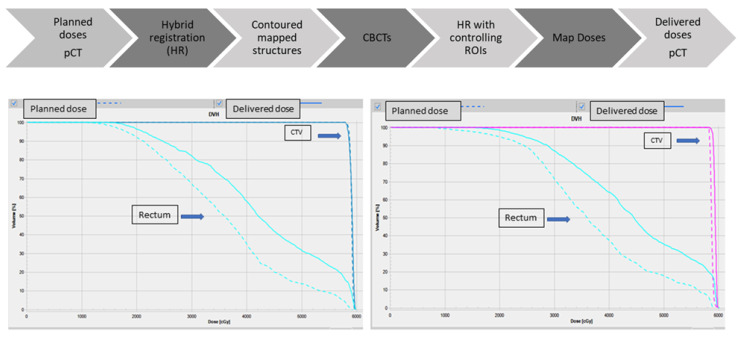
Deformable image registration ART workflow and planned and delivered dose volume histograms comparison. pCT: planning CT; ART: adaptive radiotherapy; CBCT, cone-beam computed tomography; ROI: region of interest; CTV: clinical target volume.

After that, the CTV, rectum, and bladder could be mapped from the pCT to each CBCT. Because the result of this mapping did not perfectly match the real shape and location of these structures in the CBCT, they were all revised and corrected by the same physician. These corrected structures were then used to guide a second DIR between the pCT and all the CBCTs images, and therefore to reduce the uncertainty by relating these images. After this correlation, the doses of the treatment plan calculated in each CBCT were them mapped to the pCT to obtain the administered doses to the CTV and OARs for any fraction of the treatment. Finally, the dosimetric objectives and the rectal constraints were used to check if there was a CTV underdose or a rectal overdose comparing the planned doses of the initial treatment plan and the administrated doses in the pCT. Because the prostate position is mainly related to rectal filling [17], we decided to analyze the bladder separately in a forthcoming study. In summary, our adaptive process is based on comparing the planned doses versus the administered doses in the pCT (Figure 1).

### 2.6. Hypofractionated Virtual Treatment: Planned vs. What Would Have Been Administered

After assessing the doses that would have been administered during these 15 fractions, patients were stratified according to whether the doses administered to 50% of the rectum (D50) would have exceeded their dose constraint (R50) or if the CTV would not have reached the minimum acceptable dose at 98% of the volume (D98). 

We then compared the rectal and CTV volume in the pCTs with that in the CBCTs during the 15 fractions of this virtual treatment and analyzed their possible relationship with the rectal limitations being exceeded or the minimum acceptable dose for CTV not being reached. 

Finally, we calculated the D50 variation rate (VR50) = [100 × (administered D50 − planned D50/planned D50)] and the D98 variation rate (VR98) = [100 × (administered D98 − planned D98/planned D98)] during the first 5 sessions. We also studied if during these first fractions a value of VR50 or VR98 could reliably predict the risk of rectal overdosage or underdosage of CTV.

### 2.7. Nutritional Assessment Protocol

From the time of the pCT until the end of the virtual treatments, we carried out a nutritional study to analyze anthropometric and biochemical variations and their repercussions in the planned doses. 

On pCT day, a first nutritional evaluation was performed with the following parameters: height, weight, wrist circumference, complexion, and body mass index. A blood test was also performed to determine baseline albumin and prealbumin levels. In treatment fraction 1, the previous nutritional evaluation was repeated; in fraction 6, a weight control was performed exclusively. Finally, in fraction 15, a last nutritional evaluation and blood test (with albumin and prealbumin levels) were performed.

**Figure 2:**
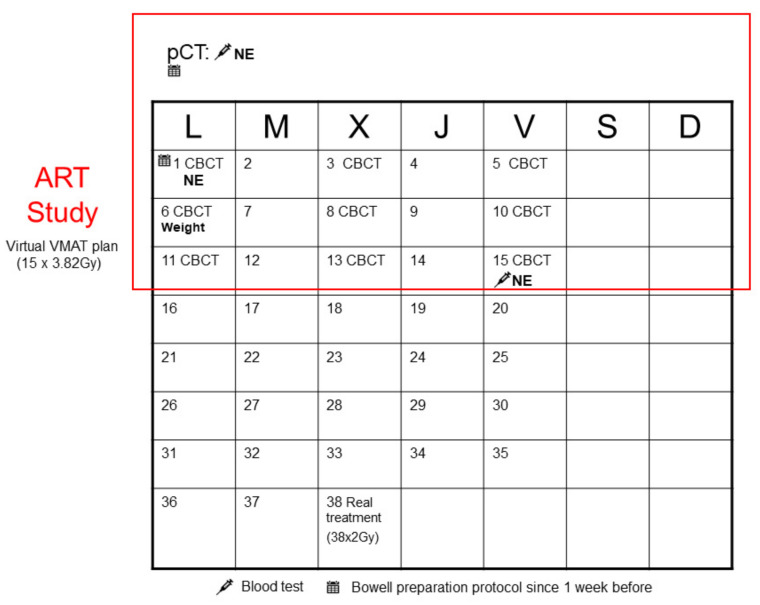
Study design. pCT: planning CT; VMAT: volumetric-modulated arc therapy; ART: adaptive radiotherapy; CBCT, cone-beam computed tomography.

All the patients started treatment on a Monday, and if there was any pause in the treatment during the first three weeks, the patient was excluded from the study. The study design is shown in Figure 2.

### 2.8. Statistical Analysis 

Quantitative variables with a normal distribution have been compared with the Student’s *t*-test, if normality was not met through the Wilcoxon signed-rank test. For the comparative analyzes by groups, one-way ANOVA were used; in case of not meeting requirements of normality and homoscedasticity, we used the Kruskal–Wallis test.

Differences between baseline and final treatment parameters have been compared with the Student’s *t*-test for paired data. For the analysis of evaluations at different moments of the study, a repeated measures ANOVA was used; in case of not fulfilling normality or homoscedasticity, we employed the Kruskal–Wallis test. Subgroup analyses were performed depending on whether the D98 CTV57 administered < 57.3 Gy, if the V51 < 20%, and according to if the V36.5 < 50%.

The area under the Receiver Operating Characteristic (ROC) curves have been used to find the patients most at risk of not complying with the planned treatment.

The expected level of significance was 5%, and the power of the statistical tests applied was 80%. The threshold for significance was *p* < 0.05. Statistical analysis was performed using IBM-SPSS 22.0 (IBM Corp; Armonk, NY, USA).

### 2.9. Clinical Correlation of the Established Predictive Values 

Our purpose was to be able to establish predictive VR50 or VR98 values that would allow us to identify a priori which patients would have excessive toxicity or inadequate tumor coverage. The problem was that they could generate doubts about their reliability because it would be based on a virtually administered treatment. However, the verification kvCBCT images were real and the anatomical variations recorded on them occurred during treatment administration. For this reason, we set out to retrospectively review which patients developed actinic proctitis or biochemical failure during follow-up. In this way, we were able to analyze whether the predictive values (VR50 and VR98) would have been able to identify the need to adapt the treatment plan in these patients, despite having been treated with different fractionation.

## 3. Results

### 3.1. Doses Administered to the Rectum 

In nine patients (37.5%), the V36.5 administered would have been higher than the limit set for it, and for the V51, the restriction would have been breached two patients. No significant differences were found between the mean V51 and V36.5 administered and those that had been planned. When we compared the mean of the rectal volume in pCTs to that of the CBCTs from sessions 1, 3, and 5, we found notable differences (*p* = 0.017, *p* = 0.015, and *p* = 0.033). 

Because the least fulfilled restriction was V36.5 < 50% and the rectal volume most significantly varies during the first five treatment sessions, we studied how the cumulative dose in 50% of the rectum (D50) evolved during this first week. We observed a significant relationship between the D50 accumulated in sessions 1, 3, and 5 and the lack of compliance with the administered R50 (Table 1). 

Finally, we performed a subgroup analysis (according to whether or not the fulfilled administered dose would have exceeded the R50 dose limitation): in fractions 1, 3, and 5, significant differences in the VR between both subgroups were found (t = −3.192, *p* = 0.008; t = −5.683, *p* < 0.001; t = −5.340, *p* < 0.001) (Table 2). In fraction 1, a value of VR > 1% would have predicted the risk of rectal overdosage with a sensitivity of 66.7% and a specificity of 73.3%. In fractions 3 and 5, a VR value > 1% would have predicted the risk of rectal overdosage in the same way, with a sensitivity of 100% and a specificity of 93.3%.

### 3.2. Doses Administered to the CTV

The average D98 administered was significantly lower than we had planned (57.55 Gy vs. 58.09 Gy, *p* = 0.026). In this structure, there were no notable differences between the mean volume of the pCT and the remaining CBCT images that could have caused this discordance. A subgroup analysis was also performed based on whether the D98 administered equaled the prescribed dose; it would have been lower in five patients and was markedly lower (by 52.02 Gy) in one compared to the others. There were no significant differences between the mean of the planned D98 and D2 PTVs for either subgroup (*p* = 0.119 and *p* = 0.80). 

Although the CTV volume did not vary significantly during the first five treatment sessions, we also studied how the cumulative D98 evolved during this first week. No significant relationship was observed between the D98 accumulated during sessions 1, 3, and 5, and the total D98 administered did not reach the minimum acceptable dose. Since there was no significant relationship between the two variables, it was not possible to establish reliable predictive values of VR98 for underdosing.

### 3.3. Anthropometric and Biochemical Variations

Significant differences were observed between the means of the pCT weight and those of the first and second week of treatment (*p* = 0.044 and *p* = 0.03). From the time of the pCT to the first therapeutic session, the patients in this cohort gained a mean of 688 g. This value progressively decreased during the second week, until the 15th session, where it reached a value very similar to that of the pCT. No correlation was found between this weight variation and the fact that the D98 and R50 administered did not meet the planned amounts (*p* = 0.093, *p* = 0.19). 

In the analyzed period (from the pCT to the 15th session), no significant variations were observed in the biochemical parameters studied (albumin, prealbumin levels, and other acute phase reactants), suggesting inflammation of the rectal mucosa.

### 3.4. Correlation of Late Rectal Toxicity and Biochemical Failure with Established Predictive Values 

After a median follow-up of 75 months, six patients developed late rectal toxicity (confirmed by colonoscopy). All episodes resolved with topical corticosteroids or endoscopic argon plasma coagulation.

Already in fraction 3 of the virtual hypofractionated treatment VR50 was able to reliably predict the risk of rectal overdose (this was probably due to the significant rectal volume variations objectified in KVCBCTs during the first week). Five of the six patients who had late rectal toxicity, in fraction 3 of the hypofractionated virtual treatment (which used their actual kvCBCT verification images), would have been considered for adaptive replanning of treatment because their RV50 was >1%. Therefore, the VR50 seems to identify rectal variations that cause the need for adapting the initial treatment plan, despite having been treated with a different fractionation. 

In the follow-up period, no biochemical recurrence was observed; moreover, it would not have been possible to study any relationship, since we were not able to find reliable predictive values of VR98 for CTV underdosage.

## 4. Discussion

A pCT is a photograph which, because of variations in the OARs during treatment, can sometimes substantially differ from the ‘anatomy of the day’ shown by the CBCT positioning image [18]. Even though we applied an intestinal preparation protocol, a significant increase in the rectal volume was observed during sessions 1, 3, and 5 compared to the volume planned for. This means that the doses administered at certain volumes were higher than had been planned. This data coincides with that published by Prabhakar et al. [19], which showed that IMRT techniques were more sensitive to rectal volumetric variations because of the higher dose gradients they create. Therefore, ART techniques are used to optimize the administration of SBRT treatments [20]. 

The strong relationship between the D50 accumulated in first fractions and the noncompliance with the R50 restriction provided us with VR values that, already in fraction 3, would very reliably alert us for sure to the risk of rectal overdosage. Only a few studies have previously demonstrated the early and specific detection with offline ART strategies of patients in which the rectum dose will exceed the tolerated limits [10]. However, because of the size of the CBCT filter, it does not always collect data for the entire rectal volume, and so these determinations should be based on estimates of doses administered only at certain rectal volumes. In this sense, our study provides useful data because it has a large sample size, uses prospective data, and was able to identify dose values administered to the entire rectal volume. In addition, the adaptive module we used in this study allowed us to calculate the cumulative dose received in any fraction [11], without having to normalize the percentage deviations of the daily doses administered.

Significant differences were found between the mean values of the D98 that were planned and those administered to the CTV. However, this does not imply that the CTV receives a subtherapeutic dose, because the mean of the D98 administered was higher than the prescribed dose. These data coincide with those from Murthy et al. [9], who observed that the proportion of the CTV that received 100% of the prescribed dose was significantly lower than had been planned. These authors also concluded that this does not imply that their treatments were incorrect. We must keep in mind that the main objective of IGRT monitoring is to ensure the dose administered to the CTV [18], which explains why only one patient received a significantly lower administered dose than the remainder of the patients. Choi et al. [8] found that the dose administered to the CTV decreases as the patient corporal contour increases, and vice versa. Thus, a corporal contour variation exceeding −1.5 cm to 2 cm is currently used to alert physicians that the treatment must be adapted. Based on this recommendation, we would have been unable to replan the treatment of the patient with the lowest D98 administered, because their body contour in session 5 varied by −1.2 cm. Of note, we did not observe a relationship between weight gain and CTV underdosage or rectal overdosage.

In our cohort, six patients developed late rectal toxicity confirmed by colonoscopy. The patients were treated with conventional fractionation (76 Gy at 38fx), but if they had been treated with the hypofractionated virtual treatment that had been studied, would they have had the same rectal toxicity? A recently published meta-analysis has shown that the moderately hypofractionated radiotherapy schedules are equal, in terms of gastrointestinal (GI) and genitourinary (GU) adverse effects, to conventional fractionation, and that the increase in GI toxicity might be related to dose escalation rather than hypofractionation [21]. 

Therefore, given these data, had we used our hypofractionated scheme, late rectal toxicity would likely have been very similar. Our ART protocol (despite being based on a different fractionation) would have identified which patients would present proctitis and would have been candidates for replanning.

In conclusion, we have developed an off-line ART protocol that can reliably identify, as early as the third session, patients with significant variations in administered doses that will lead them to exceed rectal dose constraints.

## Figures and Tables

**Table 1:** Area under ROC curves and significance for accumulated D50 in first fractions and compliance with the R50.

	Area	Asymptotic Significance	95% Asymptotic Confidence Interval	
			Lower Limit	Upper Limit
D50 accumulated fraction 1	0.841	0.006	0.682	0.999
D50 accumulated fraction 3	0.963	0.000	0.887	1.000
D50 accumulated fraction 5	1.000	0.000	1.000	1.000

D50: administered doses to the 50% of the rectum; R50: dose limitation to the 50% of the rectum established in the planned treatment.

**Table 2:** Prognostic evaluation of R50 exceeded for VR50 > 1% subgroup analysis in first fractions.

Fraction	R50 Exceeded	VR50 > 1%	Sensitivity	Specificity
	(*n*, Patients)			
1	9	6	66.7% (CI95%: 35.40–87.90)	73.30% (CI95%: 48.00–89.10)
3	9	9	100.00% (CI95%: 70.10–100.00)	93.30% (CI95%: 70.20–100.00)
5	9	9	100.00% (CI95%: 70.10–100.00)	93.30% (CI95%: 70.20–98.8)

R50: dose limitation to the 50% of the rectum established in the planned treatment; VR50: planned doses vs administered doses to the 50% of the rectum variation rate.

## Data Availability

Data are available from the authors upon reasonable request.
